# Absence of MGMT promoter methylation in diffuse midline glioma, H3 K27M-mutant

**DOI:** 10.1186/s40478-017-0500-2

**Published:** 2017-12-15

**Authors:** Rouzbeh Banan, Arne Christians, Stephan Bartels, Ulrich Lehmann, Christian Hartmann

**Affiliations:** 1 Institute of Pathology, Department of Neuropathology, Hannover Medical School (MHH), Carl-Neuberg-Str. 1, D-30625 Hannover, Germany; 2 Institute of Pathology, Hannover Medical School, Hannover, Germany

**Keywords:** Diffuse midline glioma H3 K27M-mutant, H3F3A, HIST1H3B, MGMT

**Letter**

**Fig. 1:**
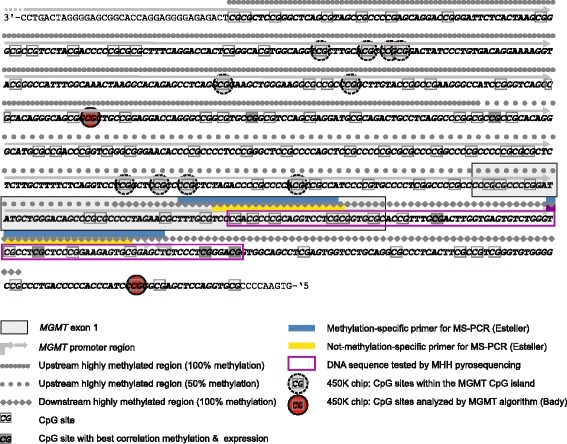
Genomic structure of the MGMT promoter. The CpG island covers the major part of the promoter region including Exon 1. The two CpG sites analyzed by the Illumina 450 K array (highlighted in red) are not associated with the DHMR as the common target region in the routinely performed assays using MS-PCR and Pyrosequencing. Our newly designed pyrosequencing assay (purple box) targets the distal section of the promoter overlapping DHMR and includes 4/6 CpG sites that highly correlate with MGMT mRNA expression

According to the revised 4th edition of the WHO classification of tumors of the CNS, diffuse midline gliomas, H3 K27M-mutant (DMG) are molecularly defined as tumors with a predominantly astrocytic differentiation carrying mutations in the histone H3 encoding genes *H3F3A* (histone H3.3), *HIST1H3B* (H3.1) or *HIST1H3C* (H3.2) [9]. The vast majority of DMG demonstrate typical features of glioblastomas WHO grade IV (GBM): Malignant astrocytic morphology, necrosis and/or microvascular proliferation. However, due to the poor clinical course of patients with DMG these tumors are assigned WHO grade IV irrespectively of GBM features. DMG are usually observed in children and young adults and occur in midline structures like thalamus, brainstem and spinal cord [9]. Most DMG carry *H3F3A* mutations; a smaller fraction shows *HIST1H3B* alterations, whereas *HIST1H3C* and, as recently shown, *HIST2H3C* mutations were identified only in single cases [10]. Around 80% of all diffuse intrinsic pontine gliomas (DIPG) exhibit the molecular profile of DMG [8]. Surgical intervention in DMG is often challenging and may lead to incomplete resection or even unsuccessful attempt failing to do a biopsy in many cases. Thus, radiotherapy and chemotherapy have a significant therapeutic importance in these patients compared with those with supratentorial GBM. Nowadays most patients with GBM receive radiotherapy and concomitant/adjuvant chemotherapy with temozolomide (TMZ) [1]. Around 40% of these cases feature hypermethylation of the promoter region of O-6-methylguanine DNA methyltransferase (*MGMT*) gene showing a positive response to TMZ treatment in comparison to those with absent *MGMT* methylation [1]. The *MGMT* gene on the chromosomal arm 10q26 consists of five exons and a CpG-rich island with 98 CpG sites covering exon 1 and most parts of the promoter (Fig. 1). Since CpG methylation pattern is not always homogenous, distinct assays may lead to conflicting results depending on the CpG sites analyzed [13]. For further understanding the contribution of each of the 98 CpG sites to MGMT expression, different studies have focused on sequencing large areas of the CpG island. By analyzing glioma cells without MGMT expression upstream and downstream highly methylated regions (UHMR, DHMR) were identified in the CpG island as well as a region in between containing a varying methylation rate (Fig. 1) [11]. Furthermore, through analyzing 52 CpG sites, the methylation status of six CpG sites was found to highly correlate with *MGMT* mRNA expression (Fig. 1) [5]. Because of the GBM-like histological appearance of most DMG, patients receive in many institutions the same treatment as those with supratentorial GBM. However, the *MGMT* promoter methylation status has not systematically been studied in patients with DMG and only few data have been reported so far [2, 3, 7, 12].

To clarify the frequency of *MGMT* promoter methylation in DMG we analyzed a retrospective series of 143 astrocytic midline tumors for *H3F3A* and *HIST1H3B* codon 27 mutations by pyrosequencing (MHH ethic board vote #1707–2013 & #6960). We identified *H3F3A* K27M mutations in 46/143 tumors including 25 males (54%) and 21 females (46%) with a median age of 23 at diagnosis and a range of 1–68 years. No *HIST1H3B* mutation was found. Next, we tested these 46 DMG for *MGMT* promoter methylation. For this purpose, DNA underwent bisulfite treatment and 14 CpG sites in the distal promoter region were analyzed by pyrosequencing (Fig. 1). A mean methylation level of 10% was defined as threshold for hypermethylation. Not a single DMG showed *MGMT* promoter hypermethylation. To compare this result with the methylation rate in none-DMG GBM we evaluated *MGMT*-promoter methylation in 40 cases of midline GBM without H3 K27M-alterations showing a hypermethylation status in 14 cases (35%) and absence of methylation in 26 tumors (65%). We, moreover, performed the same analysis in another control group of 247 patients with supratentorial GBM, IDH-wildtype that revealed hypermethylation in 94 tumors (38%) vs. 153 cases (62%) lacking *MGMT* hypermethylation.

Only few reports are available analyzing *MGMT* status in DIPG or DMG. First, Babu et al. published a series of five adult patients with DIPG. Using immunohistochemistry, they found MGMT expression in all tumors [3]. Later, the same group reported an MGMT expression frequency of 64.7% in a cohort of 34 patients with DIPG [2], while the H3 mutation status remained unknown. These findings might imply that in most adult DIPG patients the tumor carries no *MGMT* methylation. In another study, Reyes-Botero et al. found no *MGMT* promoter methylation in three adult patients with infratentorial DMG [12]. Using the Illumina 450 K methylation platform, 3/69 pediatric patients with DMG (4%) exhibited hypermethylation of the CpG site in the MGMT promoter [7]. However, only two of the 98 CpG sites of the *MGMT* CpG island are subject of analysis by the commonly applied algorithm [4] to filter 450 K data for *MGMT* promoter methylation (Fig. 1). A direct comparison between such 450 K based MGMT data and results of MS-PCR has demonstrated a good correlation [4]. Nevertheless, these two CpG sites are not located within the DHMR which is commonly analyzed by most neuropathology departments by MS-PCR or pyrosequencing [13] and has been shown to be strongly associated with the predictive role of *MGMT* promoter methylation according to the responsible CpG sites in this area (Fig. 1) [6]. To overcome this technical limitation of 450 K MGMT analysis we designed a pyrosequencing assay focusing on the DHMR [11] encompassing 4/6 CpG sites found to highly correlate with MGMT mRNA expression [5]. Therefore, we assume that the results of our study are more comparable with *MGMT* promoter methylation results of those neurooncology laboratories where 450 K technology is not available yet.

We had a median age of 23 years at diagnosis in DMP patients of our study with 9 patients aged 40 years or older including one 54- and one 68-year-old patient. These older ages of tumor occurrence in H3 K27M-mutant tumors are unusual, as these tumors have been reported to occur mostly at younger ages [10, 14].

In summary, our results and the published data clearly indicate that *MGMT* promoter methylation is a rare event in DMG patients supporting the idea that analyzing the *MGMT* promoter status would only be recommended in H3 K27M-wildtype GBM. Based on the concept that *MGMT* promoter hypermethylation is associated with a better response to TMZ through reduced expression of MGMT protein, our observation might also explain the failure of clinical trials administrating TMZ to patients with DIPG [8].
